# Prevalence of Abdominal Obesity and Metabolic Syndrome in Children and Adolescents: A Community Based Cross-Sectional Study

**Published:** 2020-02

**Authors:** Nastaran AHMADI, Seyed Mahmood SADR, Mohammad Reza MOHAMMADI, Masoud MIRZAEI, Amir Hooshang MEHRPARVAR, Seyed Mojtaba YASSINI ARDEKANI, Mohammadtaghi SAREBANHASSANABADI, Neda NILFOROSHAN, Seyed-Ali MOSTAFAVI

**Affiliations:** 1. Yazd Cardiovascular Research Center, Shahid Sadoughi University of Medical Sciences, Yazd, Iran; 2. Psychiatry and Psychology Research Center, Roozbeh Hospital, Tehran University of Medical Sciences, Tehran, Iran; 3. Department of Occupational Health Engineering, School of Public Health, Yazd University of Medical Sciences, Yazd, Iran; 4. Department of Psychiatry, School of Medicine, Shahid Sadoughi University of Medical Sciences, Yazd, Iran; 5. Department of Psychology, Yazd Azad University, Yazd, Iran

**Keywords:** Abdominal obesity, Children and adolescents, Metabolic syndrome, Prevalence

## Abstract

**Background:**

Although the prevalence of abdominal obesity and metabolic syndrome has been widely studied in the adult population, little is known about it in children and adolescents especially in developing countries. This study aimed to determine the prevalence of abdominal obesity and metabolic syndrome among children and adolescents in Yazd Greater Area, Iran; over the period of 2016–2017.

**Methods:**

This study was part of a larger national study with a cross-sectional design. Using multistage cluster random sampling method, 1035 children, and adolescents of both sexes aged 6–18 yr were randomly selected from rural and urban districts in Yazd Greater Area, Iran. Components of metabolic syndrome, and anthropometry measured in the standard situation.

**Results:**

The prevalence of abdominal obesity in children 6–10 yr old was 13.2% in boys versus 24.7% in girls. The overall prevalence of metabolic syndrome according to International Diabetes Federation (IDF) criteria in adolescents aged 10–18 yr old was 7.6% (9.4% in boys). The most prevalent metabolic syndrome components were low HDL-cholesterol (56.2%) and abdominal obesity (27.8%).

**Conclusion:**

Comparatively, the prevalence of metabolic syndrome in Yazd is high. Low HDL-cholesterol levels and abdominal obesity were the most common component, and family history of heart disease, BMI, and male gender were the main determinants of metabolic syndrome in adolescents.

## Introduction

The metabolic syndrome in children and adolescents is challenging for families, investigators, and health policy makers. At the same time, doing screen and diagnosis of this syndrome in children and adolescents are potentially more important to control chronic diseases including T2DM and cardiovascular disorders in later life (1). The diabetic and cardiovascular risk factors can be tracked from childhood to adulthood. Furthermore, metabolic syndrome brings more burden of disease in children and adolescents due to chronic characteristics of the disease (2). Hence, metabolic syndrome raises much more concerns in children and adolescents.

Diagnosis of the metabolic syndrome and estimating its prevalence in children and adolescents is controversial due to different diagnostic criteria and different ethnic, age and sex specified cut-off points (3). Abdominal obesity and low HDL-cholesterol are the most prevalent risk factors observed in most studies (4). Furthermore, abdominal obesity is a key risk factor in the diagnosis of metabolic syndrome. According to IDF criteria diagnosis of the metabolic syndrome cannot be made in children under 10 yr old, instead abdominal obesity should be reported (3).

Investigators from all around the world have reported different prevalence rates of the metabolic syndrome in adolescents among different populations and in various time frames. For instance, MacPherson, and colleagues have reported the prevalence of 2.1% among Canadian adolescents aged 10 to 18 yr (4). This rate in the United States was reported to be 4.2% to 9.2% using the Third National Health and Nutritional Survey 1988 to 1994 (5). According to IDF criteria, the prevalence of metabolic syndrome was variable from 4.1% to 6.7% in Iranian adolescents from 1990 to 2015 (6). On the other hand, when the National Cholesterol Education Program-Adult Treatment Panel III (ATP III) criteria have been applied and also among the adult population, the prevalence of metabolic syndrome is much more (7).

The metabolic syndrome components are strongly related to lifestyle; hence its prevalence is increasing in the societies with the nutritional transition and may be varied in different populations and may change during time. Although, the prevalence of metabolic syndrome has been widely studied in the adult population, still little is known about its prevalence in children and adolescents especially in developing countries. Therefore, this study aimed to determine the prevalence of abdominal obesity and metabolic syndrome among children and adolescents in Yazd Greater Area, in Iran.

## Materials and Methods

### Study Design

This was an analytical cross-sectional study which was a part of the Iranian Children and Adolescents' Psychiatric Disorders Study (IRCAP), a national project implemented in all provinces of Iran (8).

### Sampling

One thousand and thirty-five children and adolescents aged 6–18 yr were selected by multistage cluster random sampling method from Yazd greater area according to postal code during 2016–2017. The sampling method and process are fully described in the IRCAP study (9).

### Inclusion and Exclusion Criteria

Inclusion criteria were as follow: Being an Iranian citizen (residents at least for one year in Yazd district), and age range of 6 to18 yr. Children and adolescents with severe physical illnesses were excluded.

### Procedure

Trained researchers visited the participants’ home, introduced the study and described the protocol and invited the parents to participate in their children in the study after obtaining informed consent.

The interviewers collected the demographic data, as well as family history of heart disease. The criterion for family history of heart disease was having at least one first-degree relative with a diagnosis of any heart disease. Then the participants were referred to the Afshar Hospital for performing specialized tests, anthropometrics and other measurements.

In the beginning, an experienced nurse took their systolic and diastolic blood pressure three times, 15–20 min after the arrival. Blood pressure every time was taken from the right hand of the participants, in a sitting position and by using an automatic digital blood pressure device (Automatic Blood Pressure Monitor, Model M3 Comfort, Omron Co. Osaka, Japan). We used the mean of three measurements as the participant’s blood pressure. All measurements were performed in standard positions and with calibrated tools.

The nurse took venous blood from participants after 8 to 12 h of fasting for measuring fasting blood sugar and dyslipidemia including the measure of triglycerides, total cholesterol, LDL, and HDL. Then blood samples were centrifuged for serum separation. To assess fasting blood glucose and triglyceride a biochemical auto-analyzer, model BT 3000 (Italy) and PARS Azmoon Kits (Pars Azmoon Kit, Pars Azmoon Inc., Tehran, Iran) were used. Bionic kits also were used to assess high-density lipoprotein cholesterol and low-density lipoprotein cholesterol. Overall, 216 participants refused to give blood samples and at this point attrition rate was about 21%.

In addition, the nurse measured anthropometric indices including weight, height, body mass index, and waist circumference. Weight was measured using calibrated digital scale while patients were in minimal clothing and height was measured by a standard wall-height-gauge while subjects were standing without shoes and in standard position. Waist circumference was measured using a non-stretchable tape measure at the middle space between the lowest rib and the iliac crest over minimal clothing at the end of exhalation. They also recorded clinical symptoms relating to heart disease including heart palpitations, shortness of breath and chest pain in children and adolescents.

### Definition of the metabolic syndrome components

In this study we used the most consensus diagnostic criteria for the metabolic syndrome in children and adolescents from the International Diabetes Federation (IDF) (3) as follows: For children 6 to <10 yr old, the metabolic syndrome cannot be diagnosed, only ≥90^th^ percentile of waist circumference (WC) was considered for abdominal (central) obesity. We used the national age and gender specified cut-offs according to CASPIAN study (10). For children 10 to <16 yr old, the metabolic syndrome diagnosed with abdominal obesity (national cut-offs for≥90^th^ percentile of waist circumference (10)) and the presence of two or more of other criteria for metabolic syndrome ie. hypertriglyceridemia (≥150mg/dl), low HDL-chol. (<40mg/dl), high blood pressure (systolic BP≥130 or diastolic BP≥85 mm Hg), increased plasma glucose (FPG≥100mg). For children older than 16, the diagnosis of metabolic syndrome included abdominal obesity (defined as waist circumference ≥ 90^th^ percentile with ethnicity age and gender specified cut-offs for Iranians (10)) and the presence of two or more metabolic syndrome criteria’s ie. hypertriglyceridemia (≥150mg/dl), low HDL-chol. (<40mg/dl in males and <50mg/dl in females), high blood pressure (systolic BP≥130 or diastolic BP≥85 mm Hg), increased plasma glucose FPG≥100mg) or previously diagnosed T2DM.

We defined overweight as ≥95^th^ percentile of national age and sex specified cut-offs for BMI(11), at risk for overweight as ≥85^th^ to <95^th^ percentile of national age and sex specified cut-offs for BMI, and normal weight was defined as <85^th^ percentile of national age and sex specified cutoffs for BMI(12). For feasibility of comparisons between studies, we also used specified cut-off points of BMI for age and gender-based on Centers for Disease Control and Prevention 2000 (13).

### Statistical analysis

All data were analyzed using SPSS (IBM Corp. Released 2010. IBM SPSS Statistics for Windows, Ver. 19.0. Armonk, NY: IBM Corp) and STATA version 11 (StataCorp. 2009. Stata Statistical Software: Release 11. College Station, TX: StataCorp LP.). The prevalence of metabolic syndrome in children above 10 yr was measured in subgroups of gender, and BMI categories including normal weight (<85^th^ percentile), at risk for overweight (85^th^ to <95^th^ percentile), and overweight (95^th^ ≤ percentile). We used the Chi-square test to compare the prevalence of metabolic syndrome between subgroups. We also performed the logistic regression analysis to define potential predictors of the metabolic syndrome in children and adolescents above 10 yr old. In children younger than 10 yr old, the only prevalence of abdominal obesity was reported instead of the metabolic syndrome. The *P*-values below the 0.05 were considered as statistical significance.

### Ethics Statement

This study was approved by the ethics committee of National Institute for Medical Research Development (ethical code: IR.NIMAD.REC.1395.001) and Shahid Sadoughi University of Medical Sciences in Yazd, Iran (IR.SSU.Rec.1396.49).

The consent was taken from children and adolescents to participate in this study. The consent completed for participants younger than 15 yr of age by their parent and for participants aged 15 to 18 yr by parents or by the adolescents. Independent of age, all children and adolescents were assented to participate. All information about children and adolescents and their families remained confidential.

## Results

The participants of this study included 456 male (44.1%) and 579 female (55.9%). Of them, 402 (38.8%) were 6 to 9 yr and 633 (61.2%) were 10 to 18 yr old. The mean age of participants was 11.3 yr ±3.8, the mean BMI 19.5 kg/m^2^ ± 5.0 (Table 1).

**Table 1:** Characteristics of the participants in the study of prevalence of metabolic syndrome or abdominal obesity among children and adolescents of Yazd district, Iran, 2016–17

***Variable***	***Boys (mean±SD)***	***Girls (mean±SD)***	***Total (mean±SD)***
Age (yr)	11.6 ± 3.7	11.0 ± 3.9	11.32±3.8
Height (cm)	147.7±22.3	144.4±18.1	145.8±20.1
Weight (kg)	46.3±23.5	42.0±17.4	43.9±20.4
BMI (kg/m^2^)	19.8±5.5	19.2±4.6	19.5±5.0
Waist circumference (cm)	71.4±16.3	68.7±12.3	69.9±14.3
Systolic blood pressure (mmHg)	102.1±14.4	98.8±11.3	100.3±12.9
Diastolic blood pressure (mmHg)	68.5±10.0	69.3±10.0	69.0±10.0
TG (mg/dl)	87.9±46.2	88.6±37.2	88.3±41.6
HDL (mg/dl)	40.6±13.8	42.5±13.4	41.6±13.6
FBS (mg/dl)	89.5±10.0	87.4±8.4	88.4±9.2

The most prevalent component of the metabolic syndrome in our study was low HDL-cholesterol (56.2%) followed by abdominal obesity (27.8%). All of the metabolic syndrome components were highly associated with body mass index (BMI) (Table 2). According to IDF definition for metabolic syndrome, the diagnosis is valid in adolescents of 10 yr or older. In adolescents of our study with 10 to 18 yr old, the prevalence of the metabolic syndrome was 7.6% (9.4% in boys and 6.0% in girls). Alternatively, the prevalence of abdominal obesity has been reported in children younger than 10 yr; (13.2% abdominal obesity in boys vs. 24.7% in girls). Then, we have performed subgroup analysis according to BMI categories (both national and CDC cut-offs) of normal weight, at risk for overweight and over-weight and we found that prevalence of the metabolic syndrome rises with BMI (Table 3). Afterward, we entered the variables into the logistic regression model. The chance of having metabolic syndrome in male adolescents older than 10 yr was 1.6 times more compared to females (95% CI: 0.8 to 2.9). Moreover, ORs of the metabolic syndrome in adolescents older than 10 yr, respectively, were 1.7 and 5.9 for subjects at risk for overweight and overweight. The OR for the metabolic syndrome in those with a family history of heart disease was 1.58 (95% CI: 0.80 to 3.07) compared to those without the family history of heart disease.

**Table 2:** Prevalence of the individual components of metabolic syndrome among children and adolescents of Yazd district, Iran, 2016–17

***Variable***		***Abdominal obesity N(%)***	***High blood pressure N(%)***	***High blood glucose N(%)***	***Low HDL-c N(%)***	***High TG N(%)***
Sex	Total	277(27.8)	76 (8)	71(9.1)	438(56.2)	58 (7.4)
	Male	121(27.4)	37 (8.9)	41(11.2)	209(57.3)	29(7.9)
	Female	156(28.1)	39 (7.3)	30(7.2)	229(55.2)	29 (7)
BMI percentile^*^ (Iranian cut-off points)	Normal (<85^th^)	21(3.5)	35(7.6)	25(4.5)	236(51.5)	23(5)
	At risk (85^th^ to <95^th^)	72(40.7)	9(6.8)	14(8.6)	73(55.3)	12(9.1)
	Overweight (95^th^ ≤)	184(83.3)^**^	26(14.9)^*^	31(14.7)^**^	120(69)^**^	22(12.6)^*^

** Significant at *P*≤0.0001 level; using Pearson Chi-Square test (Compared with normal weight and at-risk for overweight subjects)//

* Significant at *P*≤0. 05 level; using Pearson Chi-Square test (Compared with normal weight and at-risk for overweight subjects)

**Table 3:** Prevalence of the metabolic syndrome or abdominal obesity among children and adolescents in Yazd district, Iran, 2016–17

***Variable***			***N of valid cases (excluding missing)***	***N (%) of subjects with MetS^#^***	***P (Chi-Square test)***	***N of valid cases (excluding missing)***	***N (%) of subjects with abdominal obesity***^¶^	***P (Chi-Square test)***
< 10 years	sex	Male	155	-		152	20 (13.2)	0.006
		Female	231	-		231	57(24.7)	
		Total	386	-		383	77(20.1)	
10years ≤	sex	Male	278	26(9.4)	0.1	290	101(34.8)	0.2
		Female	317	19(6.0)		325	99(30.5)	
		Total	595	45(7.6)		615	200(32.5)	
< 10 years	BMI percentile^*^	Normal (<85^th^)	238	-		238	3(1.3)	≤0.001
		At risk (85^th^ to <95^th^)	65	-		66	12(18.2)	
		Overweight (95^th^ ≤)	77	-		79	62(78.5)	
		total	380			383	77(20.1)	
10 years ≤	BMI percentile^*^	Normal (<85^th^)	357	0(0.0)	≤0.001	362	18(5)	≤0.001
		At risk (85^th^ to <95^th^)	100	10(10)		111	60(54.1)	
		Overweight (95^th^ ≤)	130	35(26.9)		142	122(85.5)	
		total	587	45(7.7)		615	200(32.5)	
< 10 years	BMI percentile^†^	Normal (<85^th^)	277	-		278	6 (2.2)	≤0.001
		At-risk (85^th^ to <95^th^)	48	-		48	21 (43.8)	
		Overweight (95^th^ ≤)	55	-		57	50 (87.7)	
		total	380			383 (100%)	77 (20.1)	
10 years ≤	BMI percentile^†^	Normal (<85^th^)	402	1(0.2)		412	33 (8)	≤0.001
		At risk (85^th^ to <95^th^)	120	19(15.8)	≤0.001	131	99 (75)	
		Overweight (95^th^ ≤)	65	25(38.5)		72	68 (94.4)	
		total	587	45(7.7)		615	200 (32.5)	

* Using Iranian data set for BMI percentiles

† Using CDC2000 data set for BMI percentiles

¶ For children 6 to <10 yr old, which according to IDF the diagnosis of metabolic syndrome cannot be made, instead, only ≥90^th^ percentile of waist circumference with national age and gender specified cut-offs

# The metabolic syndrome diagnosed for children 10 to <16 yr old, with abdominal obesity (national cut-offs for≥90^th^ percentile of waist circumference) and the presence of two or more of other criteria for metabolic syndrome i.e. hyper-triglyceridemia (≥150mg/dl), low HDL-chol. (<40mg/dl), high BP (systolic BP≥130 or diastolic BP≥85 mm Hg), increased plasma glucose FPG≥100mg). For children older than 16, abdominal obesity (wc ≥ 90^th^ percentile with ethnicity age & gender specified cut-offs for Iranians and the presence of two or more metabolic syndrome criteria’s ie. Hyper-triglyceridemia (≥150mg/dl), low HDL-chol. (<40mg/dl in males and <50mg/dl in females), high blood pressure (systolic BP≥130 or diastolic BP≥85 mm Hg), increased plasma glucose FPG≥100mg) or previously diagnosed T2DM

The OR for the metabolic syndrome in the urban regions was 1.20 (95% CI: 0.29 to 5.54) compared to the rural regions. In multivariate analysis after adjusting for gender, Family history of heart disease, Region of living, and age none of the variables remained in the model and the ORs of BMI categories were not significant (Table 4).

**Table 4:** Variables associated with metabolic syndrome upon logistic regression analysis

***Variable***	***Univariate analysis***		***Multivariate analysis***	

	***Crude OR (95% CI)***	***P-value***	Adjusted OR^*^	***P-value***
Gender	1.6 (0.8 to 2.9)	0.12		
Family history of heart disease	1.58(0.80 to 3.07)	0.18		
Region of living	1.20 (0.29 to 5.54)	0.72		
Subjects at risk for overweight	1.7 (0.41 to 6.7)	0.33	1.9 (0.21 to 5.1)	0.99
Overweight subjects	5.9 (1.3 to 8.1)	<0.001	1.1 (0.23 to 4.9)	0.99

CI: Confidence Interval

* adjusted by gender, Family history of heart disease, Region of living, and age

## Discussion

In this study, based on IDF criteria, the prevalence of the metabolic syndrome in children and adolescents aged 10 to 18 yr was 7.6% (9.4% in boys and 6% in girls) which is relatively high. Moreover, in children below 10 yr prevalence of abdominal obesity was even higher (20.1%). This is the first study reporting the prevalence of metabolic syndrome and abdominal obesity in children and adolescents in Yazd Greater Area and one of the few province-wide studies in Iran.

Worldwide, investigators have reported different prevalence rates using different diagnostic criteria. Using modified diagnosis criteria of ATP III, the prevalence of metabolic syndrome was reported in American adolescents to be 9.2% (5). The prevalence rate of 10.1% in 12 to 19 yr age group based on 2001–2010 National Health and Nutrition Examination Survey was reported in the USA (14). While usually much lower prevalence rates have been reported using IDF criteria. Different prevalence rates were reported for the metabolic syndrome in the same population (0.9% using IDF criteria versus 11.4% using de Ferranti -modified ATP III- criteria) (15). While 2.1% of metabolic syndrome were reported among Canadian adolescents aged 10 to 18 yr using IDF criteria (4).

There are a few studies investigated the prevalence of metabolic syndrome in children and adolescents in Iran. The Age-modified standards of the National Cholesterol Education Program-Adult Treatment Panel III (ATP III) criteria were used in the context of Tehran Lipid and Glucose Study. They have reported 10% of metabolic syndrome in district 13 of Tehran (16). In the framework of the CASPIAN study (2003–2004) on children and adolescents based on ATP III criteria have reported 14% of metabolic syndrome (14% in boys and 13% in girls) (17). In Ahvaz, southwest of Iran, the prevalence of metabolic syndrome has reported to be 9% (11% in boys and 7% in girls) based on modified ATP III criteria (18).

In Isfahan, 4.8% of metabolic syndrome were reported in children and adolescents using the IDF criteria (19). In framework of the Isfahan Healthy Heart Program performed from 2000 to 2007, using IDF criteria in Arak and Isfahan provinces (center of Iran) have reported the prevalence of 2.8% among girls and 6.6% among boys, while metabolic syndrome prevalence rates according to de Ferranti criteria were 14.4% among boys and 10.9% among girls (20). It also reported 5.2% metabolic syndrome in urban areas and 3.3% in rural areas using IDF criteria. Alike us, almost all of the previous reports studied, have found that metabolic syndrome mostly occurs in boys, and in urban regions (6, 14, 16–18, 20). Furthermore, some revealed gender role in this regard (21). Our study is in accord with other studies reported the abdominal obesity and low HDL-cholesterol are among the most prevalent risk factors of metabolic syndrome in children and adolescents (16, 22, 23). Poor dietary habits, low physical activity or genetic characteristics of the study population may cause this.

We have observed variations in the prevalence of metabolic syndrome in adolescents. Different definitions of the metabolic syndrome and different cut-off points explain for some of the diversity observed in the prevalence of this syndrome in different districts. Moreover, diversity in lifestyle, domestic dietary habits and different levels of physical activity can be the explanation of this diversity. Previous domestic studies reported higher metabolic syndrome have used ATP III criteria which are very similar to IDF criteria except for Rigorous cut-offs for hypertriglyceridemia (e.g. Triglyceride ≥ 110 mg/dl in ATP III vs. Triglyceride ≥ 150 mg/dl in IDF). i.e. the ATP III criteria are more inclusive.

In our study, chance of having metabolic syndrome is dramatically increased in overweight adolescents. Independent of kind of the diagnosis criteria, all previous studies have also reported such observation. Some pieces of evidence have noted that body weight disorders in childhood can be developed to eating disorders, adulthood obesity, adulthood metabolic syndrome and its related psychiatric and somatic disorders in later life (24–27). This emphasis on the priority of action against childhood obesity by family and community nutritional education and more physical activity in favor of weight management (28).

## Limitations

Lack of consensus about national growth charts and cut-off points make it hard to pick-up the best study. Furthermore, due to cross-sectional nature of this study, we can’t infer the causal relationships

## Conclusion

The prevalence of metabolic syndrome among children and adolescents in Yazd province is high compared to other studies. Low HDL-cholesterol levels and abdominal obesity were the most common component of metabolic syndrome.

The abdominal obesity was mostly observed in girls but clustering it with other components of metabolic syndrome especially lower HDL-c in males caused the overall prevalence rate of the metabolic syndrome to be higher in boys. Family history of heart disease, living in urban areas, having higher BMI and being the male gender were the main determinants of metabolic syndrome in adolescents. Hence, the researchers and health policy makers should focus to solve the puzzle of low HDL-cholesterol and high abdominal obesity in children and adolescents especially in high-risk subgroups of overweight, urban residents, and boys.

## Ethical considerations

Ethical issues (Including plagiarism, informed consent, misconduct, data fabrication and/or falsification, double publication and/or submission, redundancy, etc.) have been completely observed by the authors.

## References

[1] DeBoer MD Gurka MJ (2010). Ability among adolescents for the metabolic syndrome to predict elevations in factors associated with type 2 diabetes and cardiovascular disease: data from the national health and nutrition examination survey 1999–2006. Metab Syndr Relat Disord, 8(4):343–53. 20698802 10.1089/met.2010.0008 PMC3046372

[2] He F Rodriguez-Colon S Fernandez-Mendoza J (2015). Abdominal obesity and metabolic syndrome burden in adolescents--Penn State Children Cohort study. J Clin Densitom, 18(1):30–6. 25220887 10.1016/j.jocd.2014.07.009 PMC4314452

[3] Alberti Sir George Zimmet Paul Francine Kaufman (2007). The IDF consensus definition of the metabolic syndrome in children and adolescents. International Diabetes Federation. Available from: https://www.idf.org

[4] MacPherson M de Groh M Loukine L (2016). Prevalence of metabolic syndrome and its risk factors in Canadian children and adolescents: Canadian Health Measures Survey Cycle 1 (2007–2009) and Cycle 2 (2009–2011). Health Promot Chronic Dis Prev Can, 36(2):32–40. 26878492 10.24095/hpcdp.36.2.03 PMC4910427

[5] de Ferranti SD Gauvreau K Ludwig DS (2004). Prevalence of the metabolic syndrome in American adolescents: findings from the Third National Health and Nutrition Examination Survey. Circulation, 110(16):2494–7. 15477412 10.1161/01.CIR.0000145117.40114.C7

[6] Kelishadi R Hovsepian S Djalalinia S (2016). A systematic review on the prevalence of metabolic syndrome in Iranian children and adolescents. J Res Med Sci 21:90. 28163736 10.4103/1735-1995.192506 PMC5244691

[7] Hadaegh F Zabetian A Tohidi M (2009). Prevalence of metabolic syndrome by the Adult Treatment Panel III, International Diabetes Federation, and World Health Organization definitions and their association with coronary heart disease in an elderly Iranian population. Ann Acad Med Singapore, 38(2):142–9. 19271043

[8] Mohammadi MR Ahmadi N Kamali K (2017). Epidemiology of Psychiatric Disorders in Iranian Children and Adolescents (IRCAP) and Its Relationship with Social Capital, Life Style and Parents' Personality Disorders: Study Protocol. Iran J Psychiatry, 12(1):66–72. 28496504 PMC5425354

[9] Mohammadi MR Ahmadi N Khaleghi A (2019). Prevalence and Correlates of Psychiatric Disorders in a National Survey of Iranian Children and Adolescents. Iran J Psychiatry, 14(1):1–15. 31114613 PMC6505051

[10] Kelishadi R Gouya MM Ardalan G (2007). First reference curves of waist and hip circumferences in an Asian population of youths: CASPIAN study. J Trop Pediatr, 53(3):158–64. 17308326 10.1093/tropej/fml090

[11] Hosseini M Carpenter RG Mohammad K (1999). Standardized percentile curves of body mass index of Iranian children compared to the US population reference. Int J Obes Relat Metab Disord, 23(8):783–6. 10490777 10.1038/sj.ijo.0800924

[12] Mohammadi MR Mostafavi SA Hooshyari Z (2020). National Growth Charts for BMI among Iranian Children and Adolescents in Comparison with the WHO and CDC Curves. Child Obes, 16(1):34–43. 31599653 10.1089/chi.2019.0107

[13] Kuczmarski RJ Ogden CL Guo SS (2002). 2000 CDC Growth Charts for the United States: methods and development. Vital Health Stat 11, (246):1–190. 12043359

[14] Miller JM Kaylor MB Johannsson M (2014). Prevalence of metabolic syndrome and individual criterion in US adolescents: 2001–2010 National Health and Nutrition Examination Survey. Metab Syndr Relat Disord, 12(10):527–32. 25247821 10.1089/met.2014.0055

[15] Agudelo GM Bedoya G Estrada A (2014). Variations in the prevalence of metabolic syndrome in adolescents according to different criteria used for diagnosis: which definition should be chosen for this age group? Metab Syndr Relat Disord, 12(4):202–9. 24564686 10.1089/met.2013.0127

[16] Esmaillzadeh A Mirmiran P Azadbakht L (2006). High prevalence of the metabolic syndrome in Iranian adolescents. Obesity (Silver Spring), 14(3):377–82. 16648607 10.1038/oby.2006.50

[17] Kelishadi R Ardalan G Gheiratmand R (2006). Paediatric metabolic syndrome and associated anthropometric indices: the CASPIAN Study. Acta Paediatr, 95(12):1625–34. 17129973 10.1080/08035250600750072

[18] Rashidi H Payami SP Latifi SM (2014). Prevalence of metabolic syndrome and its correlated factors among children and adolescents of Ahvaz aged 10 – 19. J Diabetes Metab Disord, 13:53. 24860794 10.1186/2251-6581-13-53 PMC4031928

[19] Nizal Sarrafzadegan MG Sadeghi Masoumeh Nouri Fatemeh (2013). Differences in the prevalence of metabolic syndrome in boys and girls based on various definitions. ARYA Atheroscler, 9(1):70–6. 23696762 PMC3653251

[20] Ahmadi A Gharipour M Nouri F (2013). Metabolic syndrome in Iranian youths: a population-based study on junior and high schools students in rural and urban areas. J Diabetes Res, 2013:738485. 23819128 10.1155/2013/738485 PMC3683475

[21] Mohammadi MR Khaleghi A Mostafavi SA (2019). Gender Determines the Pattern of Correlation between Body Mass Index and Major Depressive Disorder among Children and Adolescents: Results from Iranian Children and Adolescents' Psychiatric Disorders Study. Child Obes, 15(5):331–7. 31070473 10.1089/chi.2018.0323

[22] Zaki ME Mohamed SK Bahgat KA (2012). Metabolic syndrome components in obese Egyptian children. Ann Saudi Med, 32(6):603–10. 23396024 10.5144/0256-4947.2012.603 PMC6081115

[23] Costa RF Santos NS Goldraich NP (2012). Metabolic syndrome in obese adolescents: a comparison of three different diagnostic criteria. J Pediatr (Rio J), 88(4):303–9. 22622762 10.2223/JPED.2200

[24] Ahmadi SM Keshavarzi S Mostafavi SA (2015). Depression and Obesity/Overweight Association in Elderly Women: a Community-Based Case-Control Study. Acta Med Iran, 53(11):686–9. 26786989

[25] Mohammadi MR Mostafavi SA Hooshyari Z (2020 ). Prevalence, correlates and comorbidities of feeding and eating disorders in a nationally representative sample of Iranian children and adolescents. Int J Eat Disord 53(3):349–361. 31742760 10.1002/eat.23197

[26] Molavi P Mikaeili N Ghaseminejad MA (2018). Social Anxiety and Benign and Toxic Online Self-Disclosures: An Investigation Into the Role of Rejection Sensitivity, Self-Regulation, and Internet Addiction in College Students. J Nerv Ment Dis, 206(8):598–605. 30020206 10.1097/NMD.0000000000000855

[27] Sadeghieh Ahari S Nikpou H Molavi P (2014). An investigation of duration of untreated psychosis and the affecting factors. J Psychiatr Ment Health Nurs, 21(1):87–92. 23590638 10.1111/jpm.12067

[28] Mostafavi SA Hosseini S (2014). Weight management, energy metabolism, and endocrine hormone. Iran J Public Health, 43(1):105–11.

